# Long-term Mortality and Hospital Readmissions Among Survivors of Sepsis in Sweden: A Population-Based Cohort Study

**DOI:** 10.1093/ofid/ofae331

**Published:** 2024-06-24

**Authors:** Malin Inghammar, Adam Linder, Maria Lengquist, Attila Frigyesi, Hanna Wetterberg, Jonas Sundén-Cullberg, Anton Nilsson

**Affiliations:** Infection Medicine, Department of Clinical Sciences Lund, Lund University, Lund, Sweden; Department of Infectious Diseases, Skåne University Hospital, Lund, Sweden; Epidemiology, Population Studies and Infrastructures (EPI@LUND), Department of Laboratory Medicine, Lund University, Lund, Sweden; Infection Medicine, Department of Clinical Sciences Lund, Lund University, Lund, Sweden; Department of Infectious Diseases, Skåne University Hospital, Lund, Sweden; Anesthesia and Intensive Care, Department of Clinical Sciences Lund, Lund University, Lund, Sweden; Department of Anesthesiology and Intensive Care, Skåne University Hospital, Lund, Sweden; Anesthesia and Intensive Care, Department of Clinical Sciences Lund, Lund University, Lund, Sweden; Department of Anesthesiology and Intensive Care, Skåne University Hospital, Lund, Sweden; Infection Medicine, Department of Clinical Sciences Lund, Lund University, Lund, Sweden; Epidemiology, Population Studies and Infrastructures (EPI@LUND), Department of Laboratory Medicine, Lund University, Lund, Sweden; Division of Infectious Diseases and Center for Infectious Medicine, Karolinska Institutet, Karolinska University Hospital Huddinge, Stockholm, Sweden; Epidemiology, Population Studies and Infrastructures (EPI@LUND), Department of Laboratory Medicine, Lund University, Lund, Sweden; Centre for Economic Demography, Lund University, Lund, Sweden

**Keywords:** epidemiology, long-term, mortality, readmission, sepsis

## Abstract

**Background:**

Survivors of sepsis may experience long-term risk of increased morbidity and mortality, but estimations of cause-specific effects beyond 1 year after a sepsis episode are lacking.

**Method:**

This nationwide population-based cohort study linked data from national registers to compare patients aged ≥18 years in Sweden admitted to an intensive care unit from 2008 to 2019 with severe community-acquired sepsis. Patients were identified through the Swedish Intensive Care Registry, and randomly selected population controls were matched for age, sex, calendar year, and county of residence. Confounding from comorbidities, health care use, and socioeconomic and demographic factors was accounted for by using entropy-balancing methods. Long-term mortality and readmission rates, total and cause specific, were compared for 20 313 patients with sepsis and 396 976 controls via Cox regression.

**Results:**

During the total follow-up period, 56% of patients with sepsis died, as opposed to 26% of the weighted controls. The hazard ratio for all-cause mortality was attenuated with time but remained elevated in all periods: 3.0 (95% CI, 2.8–3.2) at 2 to 12 months after admission, 1.8 to 1.9 between 1 and 5 years, and 1.6 (95% CI, 1.5–1.8) at >5 years. The major causes of death and readmission among the sepsis cases were infectious diseases, cancer, and cardiovascular diseases. The hazard ratios were larger among those without underlying comorbidities.

**Conclusions:**

Severe community-acquired sepsis was associated with substantial long-term effects beyond 1 year, as measured by mortality and rehospitalization. The cause-specific rates indicate the importance of underlying or undetected comorbidities while suggesting that survivors of sepsis may face increased long-term mortality and morbidity not explained by underlying health factors.

## Teaser

This nationwide cohort study examined long-term outcomes after severe community-acquired sepsis in Sweden. Mortality and readmission rates in patients with sepsis were increased and remained elevated beyond 5 years of follow-up; the dominating cause was increased burden of infectious diseases.

Sepsis is a life-threatening condition caused by a dysregulated host response to infection and is a leading cause of mortality and morbidity globally [1]. It is estimated that each year 38 million adults worldwide survive sepsis [2]. In high-income countries, awareness of sepsis has increased in recent years, and short-term mortality has decreased, leading to a growing number of patients who survive their sepsis episodes [3, 4]. It is increasingly being recognized that survivors of sepsis experience poor long-term outcomes, and numerous observational studies report that survivors may experience a range of long-term consequences, such as cognitive impairment, anxiety and depression, cardiovascular events, renal failure, and repeated episodes of infection mirrored in higher readmission rates and mortality [3]. While much attention has been focused on short-term mortality, fewer studies have explored the cause-specific long-term mortality and morbidity burden beyond 1 year [5–10].

This population-based study aimed to assess the magnitudes of the elevated cause-specific long-term mortalities and readmission rates in a cohort of patients with critically ill community-acquired sepsis in a Scandinavian setting.

## METHODS

### Study Design

We created a historical cohort based on all patients aged ≥18 years who were critically ill and admitted to an intensive care unit (ICU) with a diagnosis of community-acquired sepsis in 2008 to 2019, as identified in the Swedish Intensive Care Registry (SIR) [11], which combines individual data from several administrative health care registers. We investigated the long-term health impact of a sepsis admission on mortality and readmission rates by comparing the patients with sepsis against randomly selected controls from the background population, using an entropy-balanced design, taking into account socioeconomic and demographic characteristics, medical history and comorbidities, concomitant medication use, and health care use measures [12].

### Data Sources and Study Population

SIR is a comprehensive register that collects data from affiliated ICUs in Sweden. The coverage of SIR has expanded over the study period, with around 60% of Swedish ICUs reporting to the register in 2008 and almost 100% in 2019 [13]. The treating physician registers diagnoses in SIR according to specific guidelines to ensure high specificity [1, 14]. For the study, we defined community-acquired sepsis on admission as follows:

- Admission to the ICU ≤2 days after arrival to the emergency department or hospital ward
- Main or secondary diagnosis of sepsis according to *ICD-10* codes (R57.2, R65.1, or A41.9) or with an infection as the main diagnosis (Supplementary Table 1 for specific *ICD-10* codes)
- No prior surgical procedure as noted in SIR and no prior hospitalization 3 to 30 days before the index date
- Among individuals with more than 1 observed sepsis-related admission, only the first sepsis episode was considered.

The sepsis case definition was validated by review of the original medical records in a subset of 4764 participants admitted to 4 ICUs (2015–2017) [15]. The estimated positive predictive value according to the Sepsis-3 consensus criteria was 83%.

For each individual identified in SIR, Statistics Sweden randomly selected 20 controls from the general population, matched on sex, year of birth, and county of residence on 31 December in the year before the sepsis episode.

We obtained individual information from the following national administrative and health registers, which cover the full population of Swedish residents and can be linked with a personal identifier assigned for life to all Swedish residents: Cause of Death Register [16]; National Patient Register [17]; Prescribed Drug Register [18]; and LISA, a database managed by Statistics Sweden that contains information on socioeconomic data, mortality and migration, age, sex, area of residence, country of birth, level of education, incomes from different sources, and total disposable income per consumption unit. We also retrieved data from the National Quality Sepsis Registry (NQSR) [19]. The NQSR encompasses only hospitals where specialist infectious disease physicians are present and holds data on patients admitted to an ICU with a diagnosis of community-acquired severe sepsis or septic shock within 24 hours of arrival to an emergency department.

### Follow-up

Follow-up started at the index date of ICU admission and on the corresponding date for controls. It ended on the date of the outcome of interest, emigration, or end of study (31 December 2019), whichever came first. Outcomes were examined over different follow-ups: up to 30 days past the index date, 31 to 365 days, 1 to 3 years, 3 to 5 years, and >5 years.

Outcomes included all-cause mortality, cause-specific mortality, and cause-specific readmission during follow-up. The Swedish Cause of Death Register is a high-quality registry with almost complete data based on death certificates issued by the responsible physician. For international comparability, the underlying cause of death is automatically defined according to World Health Organization procedure [20]. An earlier validation study estimated an overall concordance of 77% between death certificate and chart review; agreement was higher in younger groups, 91% to 98% among those <65 years of age, and in disease groups such as malignant neoplasms and cardiovascular disease [21]. Despite a fall in autopsy rates, it is assumed that the quality of death certificates is increasingly high due to improvements in diagnostic practices and procedures in the last decades [16]. We created 12 groups of main diagnoses of hospitalizations and causes of death during follow-up based on the *ICD-10* chapters, except that the category of infectious diseases was expanded to include a number of codes that relate to infectious disease but belong to other chapters [22]. See Supplementary Table 1 for a complete list.

### Statistical Analysis

Hazard ratios (HRs) for all-cause and cause-specific mortality were calculated for each follow-up period by using Cox regressions with robust SE, with time since the start of the interval as the time scale variable. For all-cause mortality, we also produced Kaplan-Meier survival curves. For readmissions, we employed the multiple-events version of Cox regression proposed by Andersen and Gill and clustered SE at the individual level [23].

To control for confounding, we applied entropy balancing, a method that resembles propensity score weighting but, unlike the latter, yields perfect covariate balance across cases and controls [12, 24]. We balanced the controls to match the cases with respect to the proportions or averages of variables covering sociodemographic characteristics (age, sex, county of residence, country of birth, educational attainment, employment, and total disposable income per consumption unit), year of admission, medical history, and measures of health care use and prescription drug use, as well as interactions between all variables and an indicator for female, “younger” (<65 years), and “previously healthy” (no inpatient visits in the past 5 years and no previous health care visits or obtained pharmaceuticals among those in the analysis). In analyses of period-specific HRs, balancing was applied in the beginning of the corresponding period. Missing values were handled by including a missing category. Supplementary Table 2 provides a list of the *ICD-10* codes in the entropy-balancing models.

Analyses were performed in the subgroups of participants according to age (<65 vs ≥65 years), sex, Simplified Acute Physiology Score version 3 (SAPS3; which is calculated up to 24 hours after admission: highest, >72; middle, 61–72; lowest, ≤60), and prior health status (“previously healthy”). We also performed analyses stratified according to infectious disease site (pneumonia vs other) for the subset of patients with sepsis registered in the NQSR, where this information is available.

To further explore the validity of the case definition, sensitivity analyses were performed on patients registered in the NQSR since infectious disease specialists identified and registered these cases. Finally, we performed unadjusted analyses.

As the entropy balancing applied in the main analyses did not include interactions with registration in the NQSR, SAPS3 score, or infectious disease site, the balancing was redone (without interactions) in those subgroup analyses to ensure comparability between cases and controls.

Analyses were conducted with Stata version 17.0 (StataCorp) and the *ebalance* package [25].

## RESULTS

### Study Population

**Figure 1.**
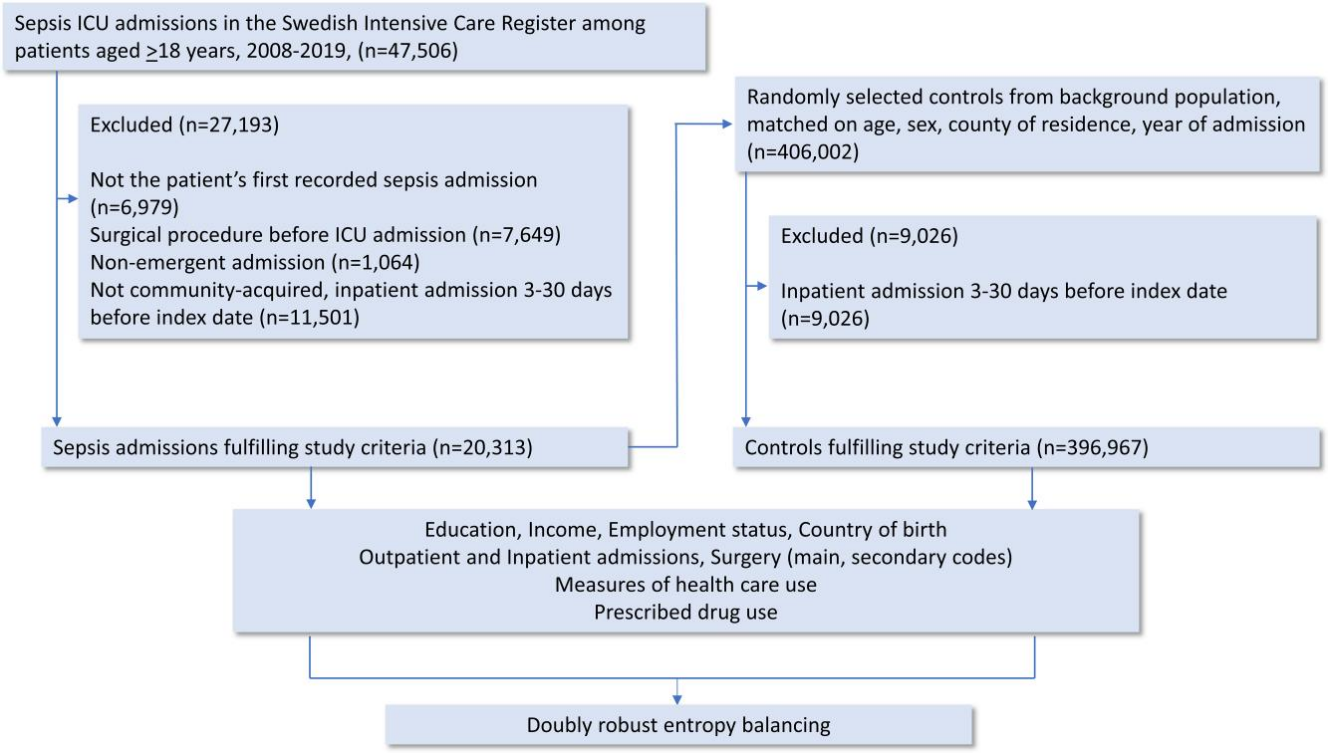
Flowchart of enrollment of sepsis admission in the Swedish Intensive Care Register (2008–2019) among patients aged ≥18 years and random selection of controls from the background population, matched for age, sex, county of residence, and year of admission. Values for exclusion criteria may not sum to the totals shown because some records were excluded for multiple reasons. ICU, intensive care unit.

Figure 1 shows the cohort selection. In the first step, 47 506 admissions with the specified *ICD-10* codes were identified in SIR. Of these, 27 193 did not fulfill the inclusion criteria, 22.1% had previous surgery or nonemergent admission, 36.2% were not deemed community acquired, and 14.7% were multiple records, leaving 20 313 in the analysis. For the controls, we excluded 2.2% who had been in inpatient care 3 to 30 days before the ICU admission to ensure comparability with the cases, leaving 396 976 controls in the final analysis.

Table 1 displays key descriptive characteristics before reweighting of the controls. As can be seen, 43% were female, and the median age was 70 years (IQR, 60–78). Patients with sepsis had lower socioeconomic status than the unweighted controls and substantially more comorbidities and higher health care use before the index. After reweighting, all variables were perfectly balanced. All controls were retained by the balancing algorithm, and virtually no controls received extreme weights: at baseline, 11 persons received weights >10, the largest weight being equal to 17; in subsequent periods, only 0 to 4 individuals received weights >10.

**Table 1.** Descriptive Statistics of the Patients With Sepsis and Unweighted Controls

	Median; Mean (IQR) or %	
Variable	Patients With Sepsis (n = 20 313)	Unweighted Controls (n = 396 976)
Basic sociodemographics		
Age, y	70; 67.49 (60–78)	70; 67.30 (60–78)
Female sex	43	43
Place of birth		
Nordic country	93	90
Non-Nordic European country	4	5
Non-European country	4	4
Education		
Missing information	2	2
Primary education	39	32
Short secondary education	29	27
Long secondary education	12	14
Tertiary education	17	26
Employment status		
Employed	19	31
Retired	63	61
Sickness absence	3	1
Unemployed	16	7
Disposable income^a^		
Quintile 1	29	20
Quintile 2	24	20
Quintile 3	19	20
Quintile 4	15	20
Quintile 5	13	20
Inpatient visits		
Past 5 y	2; 3.03 (0–4)	0; 0.89 (0–1)
Past year	0; 0.85 (0–1)	0; 0.19 (0–0)
Days, past 5 y	9; 26.76 (0–33)	0; 5.84 (0–5)
Days, past year	0; 7.79 (0–8)	0; 1.24 (0–0)
Outpatient visits		
Past year	2; 4.28 (0–5)	0; 1.53 (0–2)
Pharmaceuticals		
Types of drugs, past year	6; 6.83 (3–10)	3; 3.59 (1–6)
Inpatient visits due to infectious disease: past 5 y		
Visits	0; 0.43 (0–0)	0; 0.05 (0–0)
Days	0; 4.74 (0–0)	0; 0.51 (0.0)
Specific diagnoses or procedures: past 5 y		
Acute coronary syndrome	5	2
Other ischemic heart disease	0.2	0.1
Heart failure/cardiomyopathy	15	4
Valve disorders	5	2
Other heart disease, hypertonia, or cardiac surgery	0.4	0.2
Vascular disease	16	7
Cerebrovascular disease	8	4
Thromboembolic disease	3	1
Arrhythmia	22	11
Pulmonary disease	16	5
Rheumatic disease	5	2
Dementia	2	2
Hemiplegia or tetraplegia	2	0.2
Neurologic disease	6	2
Schizophrenia or bipolar disorder	3	1
Other psychiatric disorders	4	2
Drug, alcohol abuse, or intoxication	10	2
Diabetes	21	8
Kidney disease	10	2
Liver disease	2	0.2
Gastrointestinal disease	4	1
Neoplasms	27	18
HIV/AIDS	0.2	0.1
Immune deficiency, blood disease, or anaemia	13	3
Other (noninfectious) diseases	13	3
Enteric infection	5	1
Sepsis	8	1
Infectious of the neurologic system, including the eye	1	0.2
Upper respiratory tract infection, including the ear	4	2
Lower respiratory tract infection, including influenza	19	5
Infection		
Heart or blood vessels	1	0.1
Digestive system, including the liver	3	0.5
Genitourinary system	17	5
Skin or soft tissue	8	2
Bone, joints, or connective tissue	1	0.3
Other infections	20	6
Specific diagnoses: ever		
Cardiac surgery	12	8
Organ transplantation	2	0.3
Childhood conditions	2	0.4
Specific pharmaceuticals: last year		
Cardiac disease	70	53
Lung disease	18	10
Diabetes	22	10
Rheumatic disease	17	14
Psychiatric disease	41	23
Immunosuppressive drugs	22	7
Outcome information: general		
Follow-up time, y	1.38; 2.62 (0.05–4.35)	4.22; 4.71 (1.98–7.07)
Died during follow-up	56	15

Background characteristics refer to the status at the index date.

^a^Quintiles of disposable household income were defined according to the distribution among controls.

### Mortality

Results for cause-specific and all-cause long-term mortality are shown in Table 2, Supplementary Table 3, and Supplementary Figures 1 and 2. Patients with sepsis had a 27% 30-day all-cause mortality; the estimated mortality during the total follow-up period was 56% for patients with sepsis as opposed to 26% for the weighted controls. This in turn led to a shorter follow-up of patients with sepsis than controls (median, 1.4 and 4.2 years, respectively). The corresponding HRs for mortality were elevated in all periods although attenuated with time.

**Table 2.** Cox Regression of Major Groups for Cause-Specific Long-term Mortality: Patients With Critically Ill Sepsis vs Weighted Controls by Follow-up Period

	aHR (95% CI)				
Long-term Mortality	First Month	Months 2–12	Years 1–3	Years 3–5	Years >5
All cause	58.1 (47.6–70.9)	3.0 (2.8–3.2)	1.8 (1.7–1.9)	1.9 (1.7–2.0)	1.6 (1.5–1.8)
Infectious diseases^a^	189.5 (89.4–401.6)	6.3 (5.1–7.9)	2.1 (1.7–2.7)	2.2 (1.7–2.9)	1.9 (1.4–2.7)
Neoplasms	21.7 (14.5–32.3)	2.7 (2.4–3.1)	1.9 (1.7–2.2)	1.8 (1.6–2.1)	1.3 (1.0–1.5)
Diseases of the blood and blood-forming organs	∞^b^	3.0 (1.2–7.5)	0.8 (.2–3.3)	6.2 (2.7–14.4)	4.2 (1.6–11.0)
Endocrinologic and metabolic disorders	41.6 (23.3–72.2)	2.6 (1.9–3.5)	1.1 (.7–1.5)	2.1 (1.5–3.0)	2.2 (1.3–3.7)
Mental and behavioral disorders	17.8 (10.7–29.6)	1.6 (1.1–2.4)	1.2 (.9–1.7)	1.3 (.8–2.1)	1.2 (.8–1.7)
Nervous system disease	51.0 (29.3–88.7)	2.7 (1.9–3.9)	1.9 (1.4–2.6)	2.0 (1.4–2.8)	1.4 (1.0–2.2)
Circulatory disease	25.1 (19.4–32.6)	2.4 (2.1–2.7)	1.6 (1.4–1.7)	1.8 (1.6–2.1)	1.6 (1.4–1.9)
Respiratory disease	140.0 (63.5–308.4)	2.2 (1.6–3.0)	2.0 (1.5–2.6)	1.7 (1.1–2.8)	2.1 (1.5–2.9)
Digestive system disease	244.9 (124.0–483.7)	4.4 (2.5–7.6)	3.2 (2.4–4.4)	2.7 (1.8–3.9)	2.7 (1.8–4.2)
Musculoskeletal disease	626.0 (170.3–2301.3)	5.1 (2.4–10.6)	2.4 (1.3–4.5)	3.1 (1.0–9.2)	3.7 (1.6–8.5)
Genitourinary disease	320.1 (54.1–1895.2)	3.9 (2.4–6.1)	3.0 (2.0–4.6)	1.3 (.6–2.8)	1.9 (1.0–3.5)

aHRs were estimated by Cox regression of major groups for cause-specific long-term mortality in patients who were critically ill with sepsis and treated in an intensive care unit (2008–2019) as compared with weighted controls from the background population for different follow-up periods.

Abbreviation: aHR, adjusted hazard ratio.

^a^The category of infectious diseases was expanded to include several codes that relate to infectious diseases but belong to other chapters.

^b^Estimation not possible due to no controls with outcome.

We found substantial associations between sepsis admissions and subsequent increased mortality due to infectious disease, also in the longer term. Similar large long-term associations were found for various disease groups. In absolute numbers, patients with sepsis had high 5-year mortality risks due to infectious diseases (15% vs 3% among weighted controls), cancer (16% vs 7%), and cardiovascular diseases (18% vs 11%).

Subgroup-specific results for all-cause mortality are shown in Table 3 and in Supplementary Figure 3. Associations between sepsis episodes and long-term mortality were similar across males and females and levels of SAPS3. They were, however, stronger in younger groups than in subgroups >65 years of age and among the previously healthy than in individuals with underlying comorbidity.

**Table 3.** Cox Regression of Subgroups for All-Cause Mortality vs Weighted Controls by Follow-up Period

	aHR (95% CI)				
All-Cause Mortality	First Month	Months 2–12	Years 1–3	Years 3–5	Years >5
Sex					
Female	59.1 (41.0–85.2)	3.1 (2.7–3.5)	1.9 (1.7–2.1)	1.8 (1.6–2.1)	1.7 (1.5–1.9)
Male	57.3 (46.1–71.3)	2.9 (2.7–3.2)	1.7 (1.6–1.9)	1.9 (1.7–2.1)	1.6 (1.4–1.8)
Age, y					
<65	66.4 (28.7–153.6)	4.2 (3.2–5.5)	2.1 (1.7–2.7)	2.8 (2.1–3.7)	2.2 (1.7–2.8)
≥65	57.7 (48.5–68.7)	2.8 (2.6–3.0)	1.7 (1.6–1.8)	1.7 (1.6–1.9)	1.5 (1.3–1.6)
Previously healthy	667.2 (412.8–1078.4)	17.5 (13.4–22.7)	3.7 (2.7–5.1)	3.7 (2.6–5.2)	2.9 (2.1–3.9)
Underlying comorbidities	55.6 (45.5–68.0)	2.9 (2.7–3.1)	1.8 (1.7–1.9)	1.8 (1.7–2.0)	1.6 (1.4–1.7)
SAPS3 lowest (≤60)	25.9 (17.0–39.5)	2.6 (2.2–3.0)	1.6 (1.4–1.8)	1.7 (1.4–2.0)	1.6 (1.3–1.9)

Abbreviations: aHR, adjusted hazard ratio; SAPS3, Simplified Acute Physiology Score version 3.

### Readmission

Table 4 (recurrent-event Cox estimates) and Supplementary Figure 4 show all-cause and cause-specific rehospitalizations. The mean number of hospitalizations over a 5-year follow-up was 4.9 in sepsis cases as opposed to 3.0 among the weighted controls. Similar to the analyses of causes of death, sepsis was associated with a long-term increase in rehospitalizations for infectious disease. The most common *ICD-10* codes during follow-up were pneumonia and urinary tract infection. Subgroup-specific results for rehospitalizations are shown in Supplementary Table 4.

**Table 4.** Recurrent-Event Cox Regression of All-Cause and Cause-Specific Rehospitalization: Patients With Critically Ill Sepsis vs Weighted Controls by Follow-up Period

	aHR (95% CI)				
Rehospitalization	First Month	Months 2–12	Years 1–3	Years 3–5	Years >5
All cause	1.8 (1.7–2.0)	2.2 (2.1–2.3)	1.6 (1.5–1.7)	1.5 (1.4–1.7)	1.5 (1.3–1.6)
Infectious diseases^a^	3.7 (3.0–4.6)	3.2 (2.9–3.4)	2.4 (2.2–2.6)	2.2 (1.9–2.5)	2.1 (1.8–2.4)
Neoplasms	1.4 (1.0–1.8)	2.3 (2.0–2.6)	1.6 (1.4–1.8)	1.3 (1.0–1.5)	1.1 (.9–1.3)
Diseases of the blood and blood-forming organs	1.6 (.9–3.1)	2.4 (1.9–3.0)	1.8 (1.4–2.3)	2.0 (1.5–2.7)	1.7 (1.3–2.4)
Endocrinologic and metabolic disorders	1.8 (1.1–2.9)	2.6 (2.2–3.1)	2.1 (1.7–2.5)	2.0 (1.5–2.5)	1.9 (1.4–2.5)
Mental and behavioral disorders	0.7 (.4–1.1)	0.9 (.7–1.3)	0.9 (.6–1.4)	0.9 (.5–1.7)	0.9 (.6–1.4)
Nervous system disease	1.3 (.8–2.2)	1.9 (1.6–2.3)	1.7 (1.4–2.0)	1.6 (1.2–2.0)	1.5 (1.1–2.0)
Circulatory disease	1.2 (.9–1.5)	1.5 (1.4–1.6)	1.2 (1.1–1.3)	1.2 (1.0–1.3)	1.2 (1.0–1.4)
Respiratory disease	3.1 (1.8–5.4)	2.7 (2.2–3.2)	1.8 (1.4–2.2)	2.1 (1.6–2.7)	1.6 (1.2–2.1)
Digestive system disease	1.6 (.9–2.8)	2.6 (2.2–3.1)	1.9 (1.5–2.3)	1.7 (1.4–2.1)	1.9 (1.5–2.4)
Musculoskeletal disease	0.6 (.2–1.9)	1.1 (.7–1.6)	1.0 (.7–1.5)	1.6 (1.0–2.6)	1.1 (.6–2.0)
Genitourinary disease	2.6 (1.7–4.1)	3.0 (2.2–4.0)	2.1 (1.7–2.7)	2.1 (1.6–2.8)	2.6 (2.0–3.3)

aHRs were estimated by recurrent-event Cox regression for all-cause and cause-specific rehospitalization among patients with critically ill sepsis who were treated in an intensive care unit (2008–2019) as compared with weighted controls from the background population for different follow-up periods.

Abbreviation: aHR, adjusted hazard ratio.

^a^The category of infectious diseases was expanded to include several codes that relate to infectious diseases but belong to other chapters.

### Sensitivity Analyses

In unadjusted analyses (Supplementary Table 5), the associations were much stronger for mortality and rehospitalization as compared with the adjusted analyses.

In sensitivity analysis restricted to cases registered in the NQSR and the corresponding controls (n = 50 294), associations were very similar to the main results (Supplementary Table 6). We found no major differences in long-term survival depending on the site of infection (Supplementary Table 7).

## DISCUSSION

In this nationwide study of patients who were critically ill with community-acquired sepsis in Sweden, long-term mortality and rehospitalization rates were increased vs general population controls. The associations were attenuated with longer follow-up but remained elevated beyond 5 years. The main causes of death and readmission in sepsis cases were infectious diseases and cancer.

While some studies have reported persisting health effects for up to 1 year in survivors of sepsis, only a few have studied mortality beyond the first year as compared with controls [3, 6–10, 26–29]. In line with our results, Wang et al reported elevated HRs for all-cause mortality >5 years from a cohort of 975 patients with sepsis in the REGARDS population-based study (HR, 1.4 at >5 years) [10]. In a Canadian cohort of 1030 patients who were critically ill with sepsis, Linder et al reported a 2-fold increased 10-year mortality ratio as compared with age- and gender-standardized mortality rates from the general population [9]. In a large study from Canada of 196 000 patients with severe sepsis, Farrah et al reported HRs for mortality between 1.5 and 1.7 after 1 to 5 years as compared with propensity score–matched hospital controls [7].

Despite a significant body of evidence suggesting associations between sepsis and a range of long-term effects, such as cognitive impairment, anxiety, and cardiovascular events, it has been challenging to disentangle the causal effects [30]. Postsepsis morbidity may have multiple explanations, including underlying health status, new or aggravated comorbidities, and sequelae of the acute illness [5, 31]. Our results show that sepsis confers an additional risk in line with results from a US study by Prescott et al, which followed patients with sepsis for up to 2 years, comparing them against population and hospital controls [6]. However, our results suggest that a sepsis episode may be the first sign (indicator disease) of other serious illnesses, as stratified analyses among study participants with no prior specialized care contact and no prior filled prescriptions (ie, previously healthy) showed increased readmission rates and late mortality due to cancer up to 3 to 5 years after index. Finally, the results show that the degree of the acute illness influences the late mortality risk, as there was a clear gradient with increasing late mortality according to the SAPS3 score, although we did not find a gradient with the long-term readmission rates. The HRs were attenuated in later periods, but the greater risk persisted even beyond 5 years of follow-up. This was also demonstrated in the study by Prescott et al, where late mortality was higher in patients with more organ failure [6].

Previous research has shown that a prolonged immunosuppressive phase may follow after the initial hyperinflammatory state in sepsis [31]. In a study by DeMerle et al, half of the readmissions for sepsis within the first 3 months were deemed to be caused by new infections [32]. Dahlberg et al found that 32% of the readmissions during the first year after sepsis were due to infections, which was significantly higher than in hospital controls [33]. Furthermore, Shen et al reported an increased risk of recurrent sepsis up to 8 years after the first episode [34]. Similarly, the higher mortality and rehospitalization in our study were largely explained by the greater risk of infectious diseases. Even among the previously healthy, rehospitalization rates for infectious disease were greatly increased during the follow-up period.

The interpretation of the long-term health impact of sepsis in epidemiologic studies depends on the study population, the health care system, and the choice of control group [5]. A meta-analysis of available studies reported that the increased risk of late mortality up to 1 year after the sepsis episode was highest when compared with controls in the general population [3]. Studies of hospitalized control populations have shown more attenuated results [6]. In a study from Sweden based on the SIR database (2008–2014), Wilhelms et al found a higher 30-day mortality rate in patients with sepsis but no difference in 1-year death rates (around 53%) as compared with nonsepsis ICU cases matched on age, sex, and severity of illness according to SAPS3 score [35].

The aim of the present study was to assess the effect of sepsis on public health, applying a person-centered perspective. For this purpose, controls from the background population, carefully matched to resemble the sepsis cases with respect to comorbidity and socioeconomic background, are the most valid comparison group. Comparing the cases with patients who have other severe illnesses or are hospitalized would, however, underestimate the total morbidity burden and thus also the need for follow-up and preventive measures for the individual patients after the sepsis episode.

Our study has several strengths. First, selection bias was minimized since the Swedish health care system covers all residents. Second, information to this study was collected through national health registries with complete information and high validity, enabling long observation and no loss to follow-up [17]. Moreover, we controlled for a range of confounders using the entropy-balancing method, enabling a complete balance of covariates between patients with sepsis and controls. In sensitivity analyses, in which we did not adjust for confounders, estimates were much stronger than the main adjusted analyses, underscoring the importance of robust confounder control. Finally, we used a rigorous coding of infectious disease diagnoses. Many of these codes belong to other *ICD-10* chapters. If these codes are not regrouped, the burden of infectious diseases may be underestimated [22].

A limitation is that the study includes only patients with sepsis treated in the ICU. The results may therefore not be generalizable to patients with sepsis who are not critically ill. The sepsis case definition in our study was validated according to the Sepsis-3 consensus by review of original medical files, and 83% fulfilled the criteria. Sepsis-3 was implemented in 2018 in SIR. The older Sepsis-1 and Sepsis-2 criteria, including less complicated infections, were in use during the earlier phases of the study [14, 36]. Despite this, we believe that the specificity for severe sepsis and septic shock was high throughout the study, since only patients admitted to an ICU within 2 days after arrival at an emergency department were included. While we used robust measures for confounder control, we cannot exclude residual confounding either from unknown factors or from factors unavailable in national databases (eg, body mass index and smoking) and therefore any overestimated associations.

Another possible threat to the validity of the cause-specific mortality results is that different causes of death may act as competing events—specifically, that death from 1 cause precludes the possibility of dying from another. Similarly, death from any cause precludes the possibility of additional health care visits. These selection issues may impair the ability to interpret estimated effects as marginal effects (ie, effects that would arise if no precluding events were present) unless the different outcomes are independent [37]. While some authors, in the presence of competing events, choose to estimate subdistribution hazards where individuals are kept in the risk set even if a competing event has occurred [38], we here prefer to note competing events as a potential limitation of our analysis, as subdistribution hazards are primarily useful for prediction and are not informative of disease etiology [37, 39].

In conclusion, our study shows that severe community-acquired sepsis was associated with substantial increased mortality and rehospitalization beyond 1 year. Our results indicate the importance of underlying or undetected comorbidities for long-term outcomes, but they also suggest that survivors of sepsis may experience a higher burden of mortality and morbidity not explained by underlying health factors. It is of great importance to gain an understanding of life after sepsis and to disentangle modifiable factors [40]. Follow-up programs targeting patients at risk for repeated admissions and implementing tailored and individualized preventive measures can potentially reduce long-term disease burden.

## Supplementary Data

Supplementary materials are available at *Open Forum Infectious Diseases* online. Consisting of data provided by the authors to benefit the reader, the posted materials are not copyedited and are the sole responsibility of the authors, so questions or comments should be addressed to the corresponding author.

## Supplementary Material

ofae331_Supplementary_Data

## Notes

***Acknowledgments.*** We thank Dr Sten Walther, SIR and Linköping University Hospital, Sweden, for the thoughtful advice and help extracting data from SIR.

***Author contributions.*** M. I. was the initiator of the study. M. I. and A. N. designed the study with assistance from J. S.-C., A. L., and H. W. M. I. was responsible for data acquisition. A. N. performed the statistical analyses. A. F. and M. L. performed chart review. M. I. and A. N. constructed figures and tables. M. I. wrote the first draft. All authors critically interpreted data and reviewed the text.

***Disclaimer.*** The funders played no role in the study’s design, data collection or analysis, the decision to publish, or the preparation of the manuscript.

***Patient consent statement.*** The study was approved by the ethical review board (Etikprövningsmyndigheten, Dnr 2019-03284). Written informed consent was waived.

***Financial support*.** This work was supported by grants from the Swedish Society for Medical Research and Swedish Governmental Funds for Clinical Research.

## References

[1] Singer M, Deutschman CS, Seymour CW, et al The third international consensus definitions for sepsis and septic shock (Sepsis-3). JAMA 2016; 315:801–10. 26903338 10.1001/jama.2016.0287 PMC4968574

[2] Rudd KE, Johnson SC, Agesa KM, et al Global, regional, and national sepsis incidence and mortality, 1990–2017: analysis for the Global Burden of Disease Study. Lancet 2020; 395:200–11. 31954465 10.1016/S0140-6736(19)32989-7 PMC6970225

[3] Shankar-Hari M, Saha R, Wilson J, et al Rate and risk factors for rehospitalisation in sepsis survivors: systematic review and meta-analysis. Intensive Care Med 2020; 46:619–36. 31974919 10.1007/s00134-019-05908-3 PMC7222906

[4] Reinhart K, Daniels R, Kissoon N, Machado FR, Schachter RD, Finfer S. Recognizing sepsis as a global health priority—a WHO resolution. N Engl J Med 2017; 377:414–7. 28658587 10.1056/NEJMp1707170

[5] Prescott HC, Iwashyna TJ, Blackwood B, et al Understanding and enhancing sepsis survivorship: priorities for research and practice. Am J Respir Crit Care Med 2019; 200:972–81. 31161771 10.1164/rccm.201812-2383CP PMC6794113

[6] Prescott HC, Osterholzer JJ, Langa KM, Angus DC, Iwashyna TJ. Late mortality after sepsis: propensity matched cohort study. BMJ 2016; 353:i2375. 27189000 10.1136/bmj.i2375 PMC4869794

[7] Farrah K, McIntyre L, Doig CJ, et al Sepsis-associated mortality, resource use, and healthcare costs: a propensity-matched cohort study. Crit Care Med 2021; 49:215–27. 33372748 10.1097/CCM.0000000000004777

[8] Shankar-Hari M, Harrison DA, Ferrando-Vivas P, Rubenfeld GD, Rowan K. Risk factors at index hospitalization associated with longer-term mortality in adult sepsis survivors. JAMA Netw Open 2019; 2:e194900. 31150081 10.1001/jamanetworkopen.2019.4900 PMC6547123

[9] Linder A, Guh D, Boyd JH, Walley KR, Anis AH, Russell JA. Long-term (10-year) mortality of younger previously healthy patients with severe sepsis/septic shock is worse than that of patients with nonseptic critical illness and of the general population. Crit Care Med 2014; 42:2211–8. 25054672 10.1097/CCM.0000000000000503

[10] Wang HE, Szychowski JM, Griffin R, Safford MM, Shapiro NI, Howard G. Long-term mortality after community-acquired sepsis: a longitudinal population-based cohort study. BMJ Open 2014; 4:e004283. 10.1136/bmjopen-2013-004283 PMC3902401 24441058

[11] Sjoberg F, Walther S. Intensive care registries and the evolution of the concept of “quality of care”—reflections from the 10-year anniversary symposium of the Swedish Intensive Care Registry. Acta Anaesthesiol Scand 2012; 56:1073–7. 22967196 10.1111/j.1399-6576.2012.02757.x

[12] Hainmueller J . Entropy balancing for causal effects: a multivariate reweighting method to produce balanced samples in observational studies. Polit Anal 2012; 20:25–46.

[13] Swedish Intensive Care Registry. ÅSI. Available at: https://www.icuregswe.org/data–resultat/arsrapporter/. Accessed 13 October 2023.

[14] Levy MM, Fink MP, Marshall JC, et al 2001 SCCM/ESICM/ACCP/ATS/SIS International Sepsis Definitions Conference. Intensive Care Med 2003; 29:530–8. 12664219 10.1007/s00134-003-1662-x

[15] Lengquist M, Lundberg OHM, Spangfors M, et al Sepsis is underreported in Swedish intensive care units: a retrospective observational multicentre study. Acta Anaesthesiol Scand 2020; 64:1167–76. 32463121 10.1111/aas.13647

[16] Brooke HL, Talback M, Hornblad J, et al The Swedish Cause of Death Register. Eur J Epidemiol 2017; 32:765–73. 28983736 10.1007/s10654-017-0316-1 PMC5662659

[17] Ludvigsson JF, Andersson E, Ekbom A, et al External review and validation of the Swedish National Inpatient Register. BMC Public Health 2011; 11:450. 21658213 10.1186/1471-2458-11-450 PMC3142234

[18] Wettermark B, Hammar N, Fored CM, et al The new Swedish Prescribed Drug Register—opportunities for pharmacoepidemiological research and experience from the first six months. Pharmacoepidemiol Drug Saf 2007; 16:726–35. 16897791 10.1002/pds.1294

[19] Inghammar M, Sunden-Cullberg J. Prognostic significance of body temperature in the emergency department vs the ICU in patients with severe sepsis or septic shock: a nationwide cohort study. PLoS One 2020; 15:e0243990. 33373376 10.1371/journal.pone.0243990 PMC7771849

[20] World Health Organization. International Statistical Classification of Diseases and Related Health Problems. Available at: https://www.who.int/standards/classifications/classification-of-diseases. Accessed 13 October 2023.

[21] Johansson LA, Bjorkenstam C, Westerling R. Unexplained differences between hospital and mortality data indicated mistakes in death certification: an investigation of 1,094 deaths in Sweden during 1995. J Clin Epidemiol 2009; 62:1202–9. 19364635 10.1016/j.jclinepi.2009.01.010

[22] Torisson G, Rosenqvist M, Melander O, Resman F. Hospitalisations with infectious disease diagnoses in somatic healthcare between 1998 and 2019: a nationwide, register-based study in Swedish adults. Lancet Reg Health Eur 2022; 16:100343. 35360441 10.1016/j.lanepe.2022.100343 PMC8960944

[23] Andersen PK, Gill RD. Cox's regression model for counting processes: a large sample study. Ann Stat 1982; 10:1100–20.

[24] Watson SK, Elliot M. Entropy balancing: a maximum-entropy reweighting scheme to adjust for coverage error. Qual Quant 2016; 50:1781–97.

[25] Henmueller J, Xu Y. Ebalance: a Stata package for entropy balancing. J Stat Softw 2013; 54:7.

[26] Gritte RB, Souza-Siqueira T, Curi R, Machado MCC, Soriano FG. Why septic patients remain sick after hospital discharge? Front Immunol 2020; 11:605666. 33658992 10.3389/fimmu.2020.605666 PMC7917203

[27] Iwashyna TJ, Ely EW, Smith DM, Langa KM. Long-term cognitive impairment and functional disability among survivors of severe sepsis. JAMA 2010; 304:1787–94. 20978258 10.1001/jama.2010.1553 PMC3345288

[28] Karlsson S, Ruokonen E, Varpula T, Ala-Kokko TI, Pettila V; Finnsepsis Study Group. Long-term outcome and quality-adjusted life years after severe sepsis. Crit Care Med 2009; 37:1268–74. 19242321 10.1097/CCM.0b013e31819c13ac

[29] Fleischmann-Struzek C, Rose N, Freytag A, et al Epidemiology and costs of postsepsis morbidity, nursing care dependency, and mortality in Germany, 2013 to 2017. JAMA Netw Open 2021; 4:e2134290. 34767025 10.1001/jamanetworkopen.2021.34290 PMC8590172

[30] Munroe E, Prescott HC. Late mortality from sepsis: what we know and what it means. Crit Care Med 2021; 49:353–5. 33438972 10.1097/CCM.0000000000004795

[31] van der Slikke EC, An AY, Hancock REW, Bouma HR. Exploring the pathophysiology of post-sepsis syndrome to identify therapeutic opportunities. EBioMedicine 2020; 61:103044. 33039713 10.1016/j.ebiom.2020.103044 PMC7544455

[32] DeMerle KM, Royer SC, Mikkelsen ME, Prescott HC. Readmissions for recurrent sepsis: new or relapsed infection? Crit Care Med 2017; 45:1702–8. 28742549 10.1097/CCM.0000000000002626 PMC5600690

[33] Dahlberg J, Linder A, Mellhammar L. Use of healthcare before and after sepsis in Sweden: a case-control study. BMJ Open 2023; 13:e065967. 10.1136/bmjopen-2022-065967 PMC9944643 36806063

[34] Shen HN, Lu CL, Yang HH. Risk of recurrence after surviving severe sepsis: a matched cohort study. Crit Care Med 2016; 44:1833–41. 27120256 10.1097/CCM.0000000000001824

[35] Wilhelms SB, Walther SM, Sjoberg F, De Geer L. Causes of late mortality among ICU-treated patients with sepsis. Acta Anaesthesiol Scand 2020; 64:961–6. 32319686 10.1111/aas.13592

[36] Bone RC, Sibbald WJ, Sprung CL. The ACCP-SCCM consensus conference on sepsis and organ failure. Chest 1992; 101:1481–3. 1600757 10.1378/chest.101.6.1481

[37] Geskus RB . Competing risks: aims and methods. In: Srinivasa Rao ASR, Rao CR, eds. Handbook of statistics. Vol 43. Amsterdam: Elsevier, 2020:249–87.

[38] Fine JP, Gray RJ. A proportional hazards model for the subdistribution of a competing risk. J Am Stat Assoc 1999; 94:496–509.

[39] Austin PC, Lee DS, Fine JP. Introduction to the analysis of survival data in the presence of competing risks. Circulation 2016; 133:601–9. 26858290 10.1161/CIRCULATIONAHA.115.017719 PMC4741409

[40] Taylor SP, Bray BC, Chou SH, Burns R, Kowalkowski MA. Clinical subtypes of sepsis survivors predict readmission and mortality after hospital discharge. Ann Am Thorac Soc 2022; 19:1355–63. 35180373 10.1513/AnnalsATS.202109-1088OC PMC9353958

