# Supplementary material for: Long-term Mortality and Hospital Readmissions Among Survivors of Sepsis in Sweden: A Population-Based Cohort Study
Source: Open Forum Infect Dis. 2024 Jun 24;11(7):ofae331. doi: 10.1093/ofid/ofae331 (PMC11221654; doi:10.1093/ofid/ofae331)
Supplement: ofae331_Supplementary_Data [file ofae331_supplementary_data.pdf]

Supplementary appendix: Long-term mortality and hospital readmissions among sepsis survivors in Sweden

Malin Inghammar, Adam Linder, Maria Lenquist, Attila Frigyesi, Hanna Wetterberg, Jonas Sundén-Cullberg, Anton Nilsson

Supplementary Table 1: Codes for infectious diseases (ID-codes)

**Codes for infectious as main disease in SIR**	ICD-10: A00-B99, G00-01, G03.9, G06, I33, I38, I39.8, J01-03, J09-12, J13-16, J18, J22, J86.9, K57, K80.3, K83.0, L03-04, L08.8, L08.9, M00, M01.0, M46.2-3, M46.5, M86.0, N10, N39.0, R57.2, R65.1, T79.3, T81.4, T82.6, T82.7, T85.7).
**Codes used for outcome definitions:**	
Enteric infection	A00-A09
Sepsis (bloodstream infections)	A40-A41, R57.2, R65.0-1
Sexually transmitted infections	A50-A64, B20-B24
Infections of the neurologic system, including the eye	A39, A80-89, B30, G00-02, G03.9, G40.0-2, G04.9, G05-08, G94.0, H00.0, H03, H04.3 .H05.0, H06.1, H10.0, H10.2-3, H10.9, H13.0-1, H16.2, H16.8-9, H19.0-2, H22.0, H32.0, H44.0, H45.1
Upper respiratory tract, including the ear	H60.0-3, H60.8-9, H61.0, H62, H66-67, H70, H73.0, H75.0, H83.0, H94.0, J00-06, J34.0, J36, J39.0-1
Lower respiratory tract infections, including influenza	A15-19, A48.1, J09-22, J44.0-1, J47, J69.0, J85-86
Infections of the heart and blood vessels	I30.1, I32.0-1, I33, I38-39, I40.0, I41.0-2, I43.0, I52.0-1, I68.1, I79.0-1, I98.0-1
Infections of the digestive system including the liver (narrow definition)	B15-19, K04.4, K04.6-7, K10.2, K11.3, K12.2, K23.0-1, K61, K63.0, K65.0, K67, K75.0, K77.0, K83.0, K93.0, K93.1
Infections of the genitourinary system	N08.0, N08.8, N10, N13.6, N15.1, N15.9, N16.0, N29.0-1, N30.0, N30.9, N33.0, N34.0-1, N37, N39.0, N41.0, N41.2-3, N43.1, N45.0, N45.9, N48.1-2, N49, N51, N61, N70-74, N75.1, N76.4, N77.0-1
Infections of the skin and soft tissue	A46, L00-03, L05.0, L08, L30.3
Infections of bone, joints and connective tissue	M00-01, M46.2-5, M49.0-2, M60.0, M63.0-2, M65.0, M68.0, M71.0-1, M72.5-6, M73.0-1, M86, M90.0-2
Infectious complications	T79.3, T80.2, T81.4, T82.6-7, T83.5-6, T84.5-7,T85.7, T87.4, T88.0
Other infections	A20-28, A30-38, A42-49, A65-69, A70-79, A92-99, B00-09, B25-27, B33-99, D70.9, D73.3, E06.0, E32.1

Classification according to: Gustav T *et al.* Hospitalisations with infectious disease diagnoses in somatic healthcare between 1998 and 2019: A nationwide, register-based study in Swedish adults. *Lancet Reg Health Eur*. 2022;16:100343

Supplementary Table 2: Diagnoses, procedures, and pharmaceuticals adjusted for

	ICD-10-SE[1], ATC[2], or procedure[3] code
• **Medical history < 5 years of index**<br>• **Main and additional discharge diagnoses**<br>• **Inpatient and Outpatient Registry**	**ICD-10 codes unless otherwise specified**
Enteric infection	A00-A09
Sepsis (bloodstream infections)	A40-A41, R57.2, R65.0-1
Sexually transmitted infections	A50-A64, B20-B24
Infections of the neurological system, including the eye	A39, A80-89, B30, G00-02, G03.9, G40.0-2, G04.9, G05-08, G94.0, H00.0, H03, H04.3 .H05.0, H06.1, H10.0, H10.2-3, H10.9, H13.0-1, H16.2, H16.8-9, H19.0-2, H22.0, H32.0, H44.0, H45.1
Upper respiratory tract, including the ear	H60.0-3, H60.8-9, H61.0, H62, H66-67, H70, H73.0, H75.0, H83.0, H94.0, J00-06, J34.0, J36, J39.0-1
Lower respiratory tract infections, including influenza	A15-19, A48.1, J09-22, J44.0-1, J47, J69.0, J85-86
Infections of the heart and blood vessels	I30.1, I32.0-1, I33, I38-39, I40.0, I41.0-2, I43.0, I52.0-1, I68.1, I79.0-1, I98.0-1
Infections of the digestive system including the liver (narrow definition)	B15-19, K04.4, K04.6-7, K10.2, K11.3, K12.2, K23.0-1, K61, K63.0, K65.0, K67, K75.0, K77.0, K83.0, K93.0, K93.1
Infections of the genitourinary system	N08.0, N08.8, N10, N13.6, N15.1, N15.9, N16.0, N29.0-1, N30.0, N30.9, N33.0, N34.0-1, N37, N39.0, N41.0, N41.2-3, N43.1, N45.0, N45.9, N48.1-2, N49, N51, N61, N70-74, N75.1, N76.4, N77.0-1
Infections of the skin and soft tissue	A46, L00-03, L05.0, L08, L30.3
Infections of bone, joints and connective tissue	M00-01, M46.2-5, M49.0-2, M60.0, M63.0-2, M65.0, M68.0, M71.0-1, M72.5-6, M73.0-1, M86, M90.0-2
Other infections	A20-28, A30-38, A42-49, A65-69, A70-79, A92-99, B00-09, B25-27, B33-99, D70.9, D73.3, E06.0, E32.1
Acute coronary syndrome (MI)	I20.0, I21-22, I23
Other ischemic heart disease	I20.1-9, I24-25
Heart failure	I11.0, I13.0,I13.2, I42.0-5, I42.7-9. I.43, I50, I51.7, J81, K76.1
Valve disorders	I05-I09, I34-37,
Other heart disease, hypertonia, cardiac surgery	I10, I11.9, I12.9, I13.9, I15, I27.1, I27.9, I30.0, I30.8-9, I51.0-6, I51.8-9
Vascular disease	I65, I70-72, I73.1, I73.8-9, I74, I77.1, I77.6, I79.0, I79.2, K55, I87.2, I89.0, I97.2, R02, Z95.1-9
Cerebrovascular disease	G45-46, I60-64, I67, I69
Tromboembolic disease	I26, I82
Arrhythmia	I44-49 (not I46.1), R00, Z45.0, Z95.0, T82.1
Pulmonary disease	E84, I27.0, I27.2-9, I28.0, I28.8-9, J41-47, J60-J67, J68.4, J70.1, J70.3, J80, J84, J92, J95, J96, J98.2, R09.2, Z99.2

Rheumatic disease	I00-02, J99.0-1, M05-09, M12.3, M13, M30, M31.1, M31.3-9, M32-34, M35.0-3, M35.8-9, M45-46, D86
Dementia	F00-03, F05.1, G30, G31.1, G31.8-9, G32
Hemiplegia, tetraplegia	G11.4, G80-82, G83.0-3, G83.8
Neurologic disease	G10-14,G20-21, G23, G35-37, G60-62, G70-71, G91, G93.1
Schizophrenia, bipolar disorder	F20-31
Other psychiatric disease	F04, F05.0, F05.8-9. F06-07, F09, F33-39, F44.9,
Drug or alcohol abuse, incl. intoxication	F10-19, K29.2, K70.0-1, G31.2, G62.1, G72.1, I42.6, R78.1-5, T40, T51, Z71.4-5
Diabetes	E10-14, I79.2
Kidney disease	N00-01, N03-07, N11-12, N17-19, N25.0, N26-27, N28.0, I12.0, I13.1, Q61.1-4, Z49, Z99.2, *Procedure codes:* DR016, DR024
Liver disease	K70.2-4, K70.9, K71.1, K71.3-5, K71.7, K72-74, K75.2-9,K76.0, K76.2-9, R18, I81, I85, I86.4, I98.2, I98.8
Gastro-intestinal disease	K50-52
Cancer, neoplasms	C00-D48, J70.0, T45.1, Z51.0, Z51.1 (*not included*: Z85)
HIV/AIDS	B20-24, F02.4, O98.7, R75, Z11.4, Z21.9, Z71.1
Immune deficiency, blood disease, anemia	D50.1-9, D51-53, D60-61, D64.9, D65-68, D69.1, D69.3-6, D70-72, D73.0-2, D73.5-9, D76, D80-84, D89
Other conditions	G96.0, E22.2, E40-44, E46, E64, E66, E86, E87, R40.2, R64, R63.4
• **Medical history at any time < 5 > years**<br>• **Main and additional discharge diagnoses**<br>• **Inpatient and Outpatient Registry**	
Any medical history	
Cardiac surgery	*Procedure codes:* F(A-X), I97.0-1,
Organ transplantation	Z94, T86, *Procedure codes:* KAS, FQA, FQB, JJC, GDG, JLE
Childhood conditions	F71.1, F72-73, F79.1, F83. F84, G80, Q01-03, Q05, Q20-26, Q90
• **ATC-codes < 1 year**<br>• **Drug prescription Registry**	
Cardiac disease	C09A-D, CCB, C08C, C08D, C03C, C03EB, C03A, C03B, C03D, C03EA, C07, C01B, C01AA05, C01DA, B01AC, B01AA, C10
Lung disease	R03AC, R03BB, R03BA, R03AK, R03DA
Diabetes	A10B A10A
Rheumatic disease	M01A
Psychiatric disease	N05A, N05B, N05C, N06A, N06D, N07BB, N07BC
Immune suppressive drugs	H02AB, L01B, L04A, L01
• **Health care use**	

No of hospitalizations in last 5 years	
No of days in hospital in last 5 years	
No of (all codes) hospitalizations in last year	
No of (all codes) outpatient visits in last year	
No of drug used in last year	
No of days of hospitalization for infectious diseases in last 5 years	
No of hospitalizations for infectious diseases in last 5 years	
No of hospitalizations in last 6 months	

1) https://www.socialstyrelsen.se/statistik-och-data/klassifikationer-och-koder/icd-10/
2) https://www.whocc.no/atc_ddd_index/
3) https://www.socialstyrelsen.se/statistik-och-data/klassifikationer-och-koder/kva/

Supplementary Table 3: All-cause mortality

	n at risk		Mortality %		aHR (95% CI)
**Period**	**Case**	**Control**	**Case**	**Control**	
First month	20,313	396,967	27	0.5	58.1 (47.6-70.9)
Month 2-12	14,682	392,104	16	6.4	3.0 (2.8-3.2)
Year 1-3	11,089	345.525	16	11	1.8 (1.7-1.9)
Year 3-5	7,040	250,569	14	9.4	1.9 (1.7-2.0)
Year 5+	4,268	168,764	19	15	1.6 (1.5-1.8)

Supplementary Figure 1:

Adjusted HRs for all-cause and cause-specific long-term mortality estimated by Cox regression in critically ill sepsis patients treated in ICU 2008-2019, compared to weighted controls from the background population, for different time periods of follow up.

The category of infectious diseases was expanded to also include a number of codes that relate to infectious disease but belong to other chapters. Abbreviations: CI, confidence interval; HR hazard ratio.

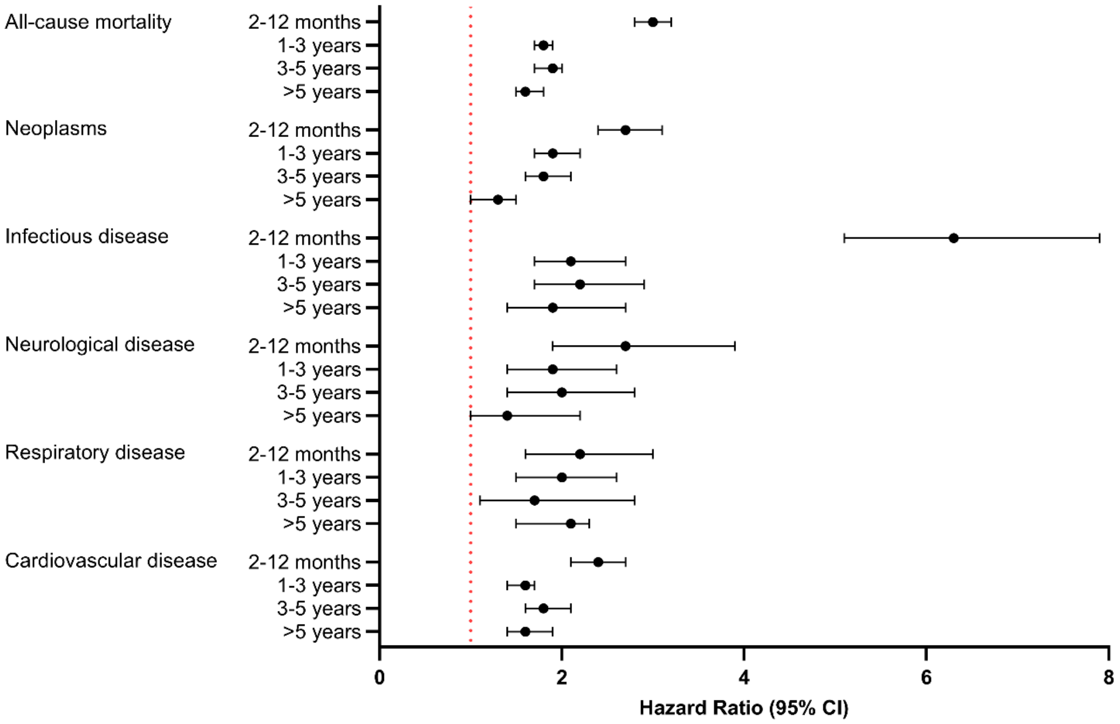

Supplementary figure 2: Kaplan-Meier graph of all-cause mortality

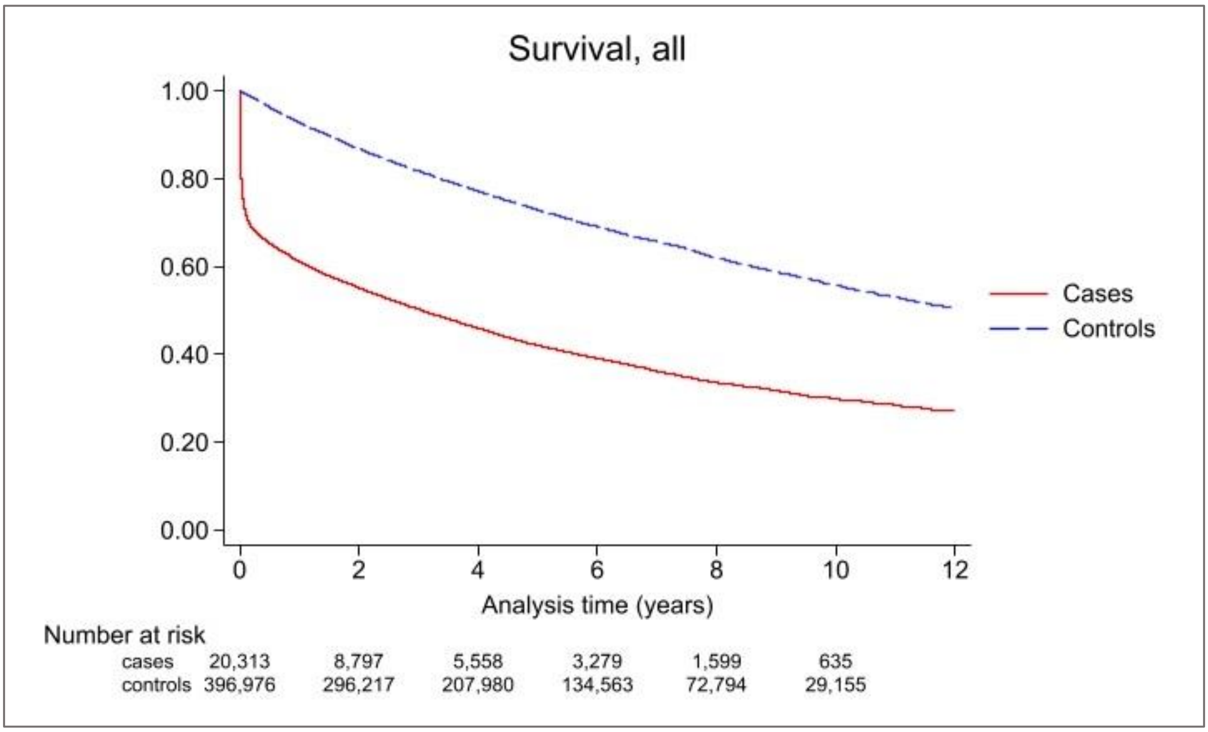

Supplementary figure 3: Kaplan-Meier graphs of all-cause mortality according to subgroups

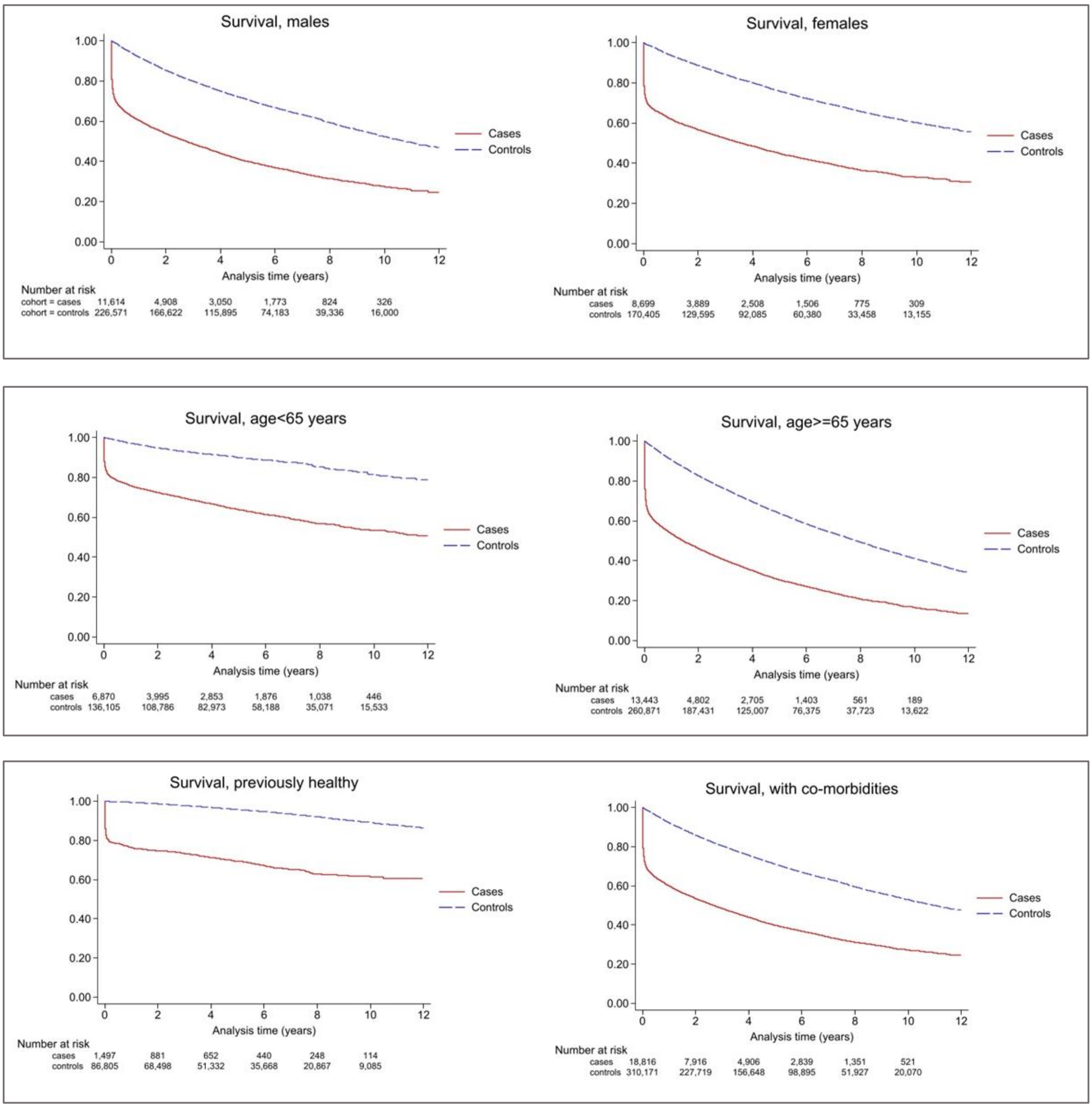

Supplementary Figure 4:

Adjusted HRs for all-cause and cause-specific re-hospitalization estimated by recurrent event Cox regression in critically ill sepsis patients treated in ICU 2008-2019, compared to weighted controls from the background population, for different time periods of follow up.

The category of infectious diseases was expanded to also include a number of codes that relate to infectious disease but belong to other chapters. Abbreviations: CI, confidence interval; HR hazard ratio.

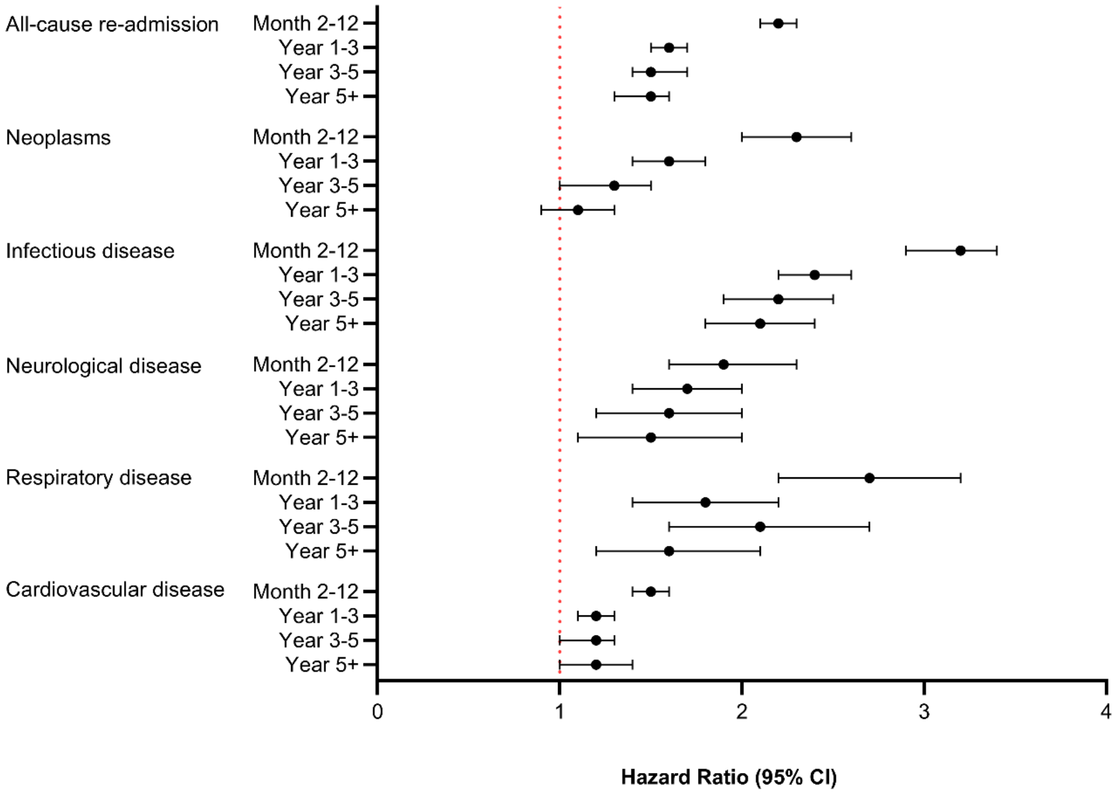

Supplementary Table 4: Subgroup-specific aHRs for rehospitalizations compared to weighted controls, estimated by recurrent-event Cox regression, for different time periods of follow up.

Rehospitalization according to subgroups	First month aHR (95% CI)	Month 2-12 aHR (95% CI)	Year 1-3 aHR (95% CI)	Year 3-5 aHR (95% CI)	Year 5+ aHR (95% CI)
Female	1.9 (1.7-2.2)	2.3 (2.2-2.5)	1.6 (1.5-1.8)	1.6 (1.5-1.8)	1.5 (1.3-1.7)
Male	1.9 (1.7-2.1)	2.1 (2.0-2.3)	1.6 (1.5-1.8)	1.5 (1.3-1.7)	1.5 (1.3-1.7)
Age <65	2.3 (1.8-2.9)	2.5 (2.2-2.8)	1.8 (1.5-2.1)	1.7 (1.4-2.1)	1.7 (1.4-2.0)
Age >65	1.8 (1.6-1.9)	2.1 (2.0-2.2)	1.5 (1.4-1.6)	1.4 (1.3-1.5)	1.3 (1.2-1.4)
Previously healthy	15.8 (12.5-20.0)	9.0 (7.9-10.1)	3.5 (2.9-4.1)	3.1 (2.6-3.7)	2.5 (2.0-3.0)
Underlying co-morbidities	1.8 (1.7-2.0)	2.2 (2.0-2.3)	1.6 (1.5-1.7)	1.5 (1.4-1.6)	1.4 (1.3-1.6)
SAPS3 Lowest (<60)	2.3 (1.9-2.8)	2.2 (2.0-2.4)	1.6 (1.4-1.8)	1.4 (1.2-1.6)	1.4 (1.2-1.7)
SAPS3 Middle (61-72)	1.9 (1.7-2.2)	2.2 (2.0-2.3)	1.6 (1.5-1.8)	1.6 (1.5-1.8)	1.6 (1.4-1.8)
SAPS3 Highest (>72)	1.3 (1.1-1.5)	2.3 (2.2-2.5)	1.7 (1.6-1.9)	1.7 (1.5-1.9)	1.3 (1.1-1.5)

Supplementary Table 5: Unadjusted and adjusted (with outpatient visits during the past 1-30 days added to the comorbidity adjustments) HRs for all-cause mortality and rehospitalizations compared to weighted controls, estimated by Cox regression, for different time periods of follow up.

	First month aHR (95% CI)	Month 2-12 aHR (95% CI)	Year 1-3 aHR (95% CI)	Year 3-5 aHR (95% CI)	Year 5+ aHR (95% CI)
**Crude-unadjusted analyses**					
All-cause mortality	183.2 (168.2-199.5)	7.4 (7.1-7.8)	3.2 (3.0-3.4)	2.6 (2.5-2.8)	1.9 (1.7-2.0)
All-cause rehospitalization	5.7 (5.4-6.1)	5.6 (5.5-5.8)	3.6 (3.5-3.7)	3.0 (2.8-3.1)	2.4 (2.3-2.5)
**Fully adjusted (incl. outpatient visits past 1-30 days)**					
All-cause mortality	58.9 (48.4-71.7)	3.0 (2.7-3.2)	1.8 (1.7-1.9)	1.9 (1.7-2.0)	1.6 (1.5-1.8)
All-cause rehospitalization	1.8 (1.7-2.0)	2.2 (2.1-2.3)	1.6 (1.5-1.7)	1.5 (1.3-1.6)	1.5 (1.3-1.6)

Supplementary Table 6: Adjusted HRs for all-cause mortality and rehospitalizations compared to weighted controls, estimated by Cox regression, for different time periods of follow up, according to registration in National Quality Register for Sepsis (NQSR).

	First month aHR (95% CI)	Month 2-12 aHR (95% CI)	Year 1-3 aHR (95% CI)	Year 3-5 aHR (95% CI)	Year 5+ aHR (95% CI)
**All-cause mortality**					
Registered in NQSR	46.2 (23.8-89.6)	2.7 (2.2-3.3)	1.8 (1.5-2.1)	1.9 (1.5-2.3)	1.5 (1.1-2.0)
Registered in SIR only	61.4 (51.2-73.5)	3.1 (2.9-3.3)	1.8 (1.7-1.9)	1.8 (1.7-2.0)	1.6 (1.5-1.8)
**All-cause rehospitalization**					
Registered in NQSR	2.1 (1.7-2.6)	2.0 (1.7-2.3)	1.6 (1.4-1.9)	1.5 (1.2-1.9)	1.4 (1.1-1.7)
Registered in SIR only	1.9 (1.7-2.0)	2.2 (2.1-2.4)	1.6 (1.5-1.8)	1.5 (1.4-1.7)	1.5 (1.3-1.6)

Supplementary Table 7: Adjusted HRs for all-cause mortality according to site of infection, compared to weighted controls, estimated by Cox regression, for different time periods of follow up, for sepsis cases registered in the National Quality Register for Sepsis (NQSR).

All-cause mortality according to site of infection	First month aHR (95% CI)	Month 2-12 aHR (95% CI)	Year 1-3 aHR (95% CI)	Year 3-5 aHR (95% CI)	Year 5+ aHR (95% CI)
Pneumonia	87.7 (41.3-186.0)	2.4 (1.7-3.5)	1.6 (1.1-2.4)	1.8 (1.2-2.6)	1.9 (1.2-3.1)
Urinary tract infection	16.8 (5.4-52.7)	2.2 (1.6-3.2)	1.8 (1.3-2.5)	1.3 (0.8-2.0)	1.2 (0.6-2.3)
Other sites of infection	57.8 (22.2-150.2)	3.8 (2.7-5.4)	1.9 (1.4-2.6)	1.9 (1.2-3.0)	2.0 (1.2-3.3)
Missing information	52.4 (15.5-177.4)	4.8 (2.2-10.5)	2.5 (1.4-4.4)	2.0 (0.9-4.7)	-
